# Is routine karyotyping required in prenatal samples with a molecular or metabolic referral?

**DOI:** 10.1186/1755-8166-5-7

**Published:** 2012-01-27

**Authors:** Angelique JA Kooper, Jacqueline JPM Pieters, Brigitte HW Faas, Lies H Hoefsloot, Ineke van der Burgt, Hans A Zondervan, Arie PT Smits

**Affiliations:** 1 Department of Human Genetics, Radboud University Nijmegen Medical Centre, Nijmegen, the Netherlands; 2 Department of Obstetrics and Gynaecology, Radboud University Nijmegen Medical Centre, Nijmegen, the Netherlands; 3 Obstetrics and Gynaecology, Rijnstate Hospital Arnhem, the Netherlands

**Keywords:** DNA diagnostics, karyotyping, mutation detection, QF-PCR, rapid aneuploidy test, prenatal diagnosis

## Abstract

As a routine, karyotyping of invasive prenatal samples is performed as an adjunct to referrals for DNA mutation detection and metabolic testing. We performed a retrospective study on 500 samples to assess the diagnostic value of this procedure. These samples included 454 (90.8%) chorionic villus (CV) and 46 (9.2%) amniocenteses specimens. For CV samples karyotyping was based on analyses of both short-term culture (STC) and long-term culture (LTC) cells. Overall, 19 (3.8%) abnormal karyotypes were denoted: four with a common aneuploidy (trisomy 21, 18 and 13), two with a sex chromosomal aneuploidy (Klinefelter syndrome), one with a sex chromosome mosaicism and twelve with various autosome mosaicisms. In four cases a second invasive test was performed because of an abnormal finding in the STC. Taken together, we conclude that STC and LTC karyotyping has resulted in a diagnostic yield of 19 (3.8%) abnormal cases, including 12 cases (2.4%) with an uncertain significance. From a diagnostic point of view, it is desirable to limit uncertain test results as secondary test findings. Therefore, we recommend a more targeted assay, such as e.g. QF-PCR, as a replacement of the STC and to provide parents the autonomy to choose between karyotyping and QF-PCR.

## Background

Currently, there is no evidence available in the literature indicating that the prevalence of chromosomal abnormalities is higher in pregnancies with a referral for DNA mutation or metabolic testing. Although the European cytogenetic guidelines for prenatal diagnosis [1] indicate that both DNA mutation and metabolic testing do not serve as referral categories for traditional karyotyping (TK), most prenatal centres worldwide routinely offer TK as an additional test. In clinical practice, most couples referred for DNA mutation analysis also opt for TK [2].

It can be disputed, however, whether TK is required when there is no *a priori *increased risk for chromosomal anomalies as compared to the normal population. On the other hand, it has been argued that when a risky invasive prenatal test is performed anyway, it is unethical not to concomitantly exclude the occurrence of putative chromosomal abnormalities [3]. With TK, a wide range of chromosomal abnormalities can be detected, including alterations in copy number (aneuploidy) and structural chromosomal rearrangements such as translocations and inversions, being either balanced or unbalanced. Targeted PCR-based assays such as multiplex ligation-dependent probe amplification (MLPA) or quantitative fluorescent PCR (QF-PCR), are highly suited for rapid aneuploidy detection (RAD) of the chromosomes 21, 18, 13, X and Y [4-12]. Previously, it has been suggested that if the referral reason is an increased risk of Down's syndrome, resulting from a positive screening test result or an advanced maternal age, karyotyping could effectively be replaced by RAD, provided that no structural fetal abnormality has been detected upon ultrasound examination [5,13-17]. The use of RAD as a targeted, standalone test instead of karyotyping when invasive prenatal testing is performed in cases with DNA mutation or metabolic test referrals, has not been studied before. This retrospective study addresses the clinical impact of TK for samples offered for prenatal diagnosis with a molecular or metabolic referral.

## Methods

The data in this retrospective study were obtained from the patient database of the Department of Human Genetics, Radboud University Nijmegen Medical Centre, the Netherlands. All procedures were performed with ethical approval from the local ethical committees. In the period January 1994 to July 2010, 500 samples from pregnant women undergoing chorionic villus (CV) sampling or amniocentesis were examined. The reason for invasive diagnostic testing was a referral for fetal DNA mutation detection, metabolic diagnostics or other "non-cytogenetic" reasons, with or without advanced maternal age (AMA). The samples were from hospitals participating in the Network Prenatal Diagnostics Nijmegen (NPDN): Radboud University Nijmegen Medical Centre, Rijnstate Hospital Arnhem, St. Elisabeth Hospital and TweeSteden Hospital Tilburg, Medical Spectrum Twente Enschede, Jeroen Bosch Hospital 's-Hertogenbosch, and some other hospitals in the Netherlands.

CV samples were split into two portions, one for DNA mutation or metabolic analysis and one for TK. On CV samples, both short-term cultures (STC) and long-term cultures (LTC) were performed. Amniotic fluid (AF) cells were cultured for TK and, simultaneously, for DNA isolation. DNA mutation analyses were performed in different diagnostic centres in the Netherlands. For metabolic testing, (cell-free) AF was used.

Karyotyping was performed following standard procedures. The results were reviewed retrospectively and classified as normal or abnormal. Additional or follow-up studies, such as parental karyotyping in case of a structural rearrangement or the presence of a marker chromosome, and fluorescence in situ hybridization (FISH) in case of insufficient test results, were performed before a definite prenatal karyotype result and its interpretation were reported. In some cases a second prenatal invasive procedure was performed. The test results of DNA mutation and metabolic testing were retrieved from the patient's records.

## Results

**Figure 1:**
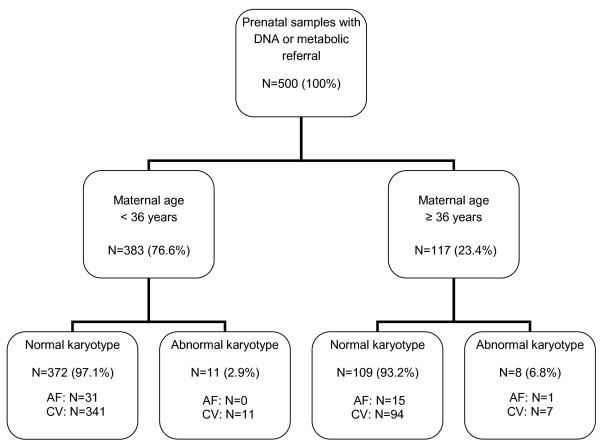
**Flow diagram for karyotyping of 500 prenatal samples**.

**Figure 2:**
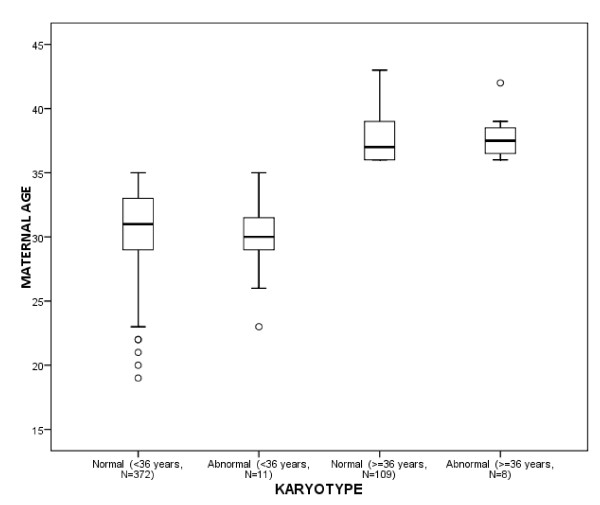
**Boxplot showing the maternal age distribution in normal and abnormal karyotype results in the groups younger or 36 years and older**. The box plots illustrate the median, 25th, and 75th percentiles. o Value more than 1.5 box-lengths from 75th/25th percentile (outliers).

The prenatal test results of 454 (90.8%) chorionic villus (CV) and 46 (9.2%) amniotic fluid (AF) samples (500 in total) were assessed. DNA mutation analysis referrals classified in normal and abnormal karyotypes are shown in Table 1. Overall, 481 (96.2%) normal karyotypes (46, XX or 46, XY) were found. The most common reasons for DNA testing were Fragile X syndrome and Huntington disease (11.4% and 7.8%, respectively). Overall, TK resulted in 19 (3.8%) abnormal karyotypes (18 CV samples and 1 AF sample). The maternal age at sampling was ≥ 36 years in 117 (23.4%) samples and resulted in 8 (6.8%) abnormal karyotypes (mean maternal age 37.9 years, median 37.5 years). The maternal age at sampling was < 36 years in 383 (76.6%) samples, and resulted in 11 (2.9%) abnormal karyotypes (mean maternal age 29.9 years, median age 30.0 years) (Figures 1 and 2). The 19 cytogenetic abnormal samples are listed in Tables 2 and 3. These included all, except one, CV samples: 4 cases with a common aneuploidy (trisomy 21, 18 and 13), 2 cases with a sex chromosomal aneuploidy (Klinefelter syndrome), one sex chromosomal mosaicism in amniotic fluid cells and different autosomal mosaicisms in 12 CV samples. These latter samples included four aberrations present in STC but absent in LTC, 4 aberrations present in LTC but absent in STC and 4 aberrations present in both STC and LTC.

**Table 1:** Ranking of diseases for DNA mutation analysis and karyotyping

Disease	N	Normal karyotype	≥ 36 years	Abnormal karyotype	Abnormal karyotype (≥ 36 years)	**Case no**.
				(< 36 years)		
Fragile X syndrome	57	57	9			
Huntington's disease	39	37	6	2		1,9
Duchenne muscular dystrophy	33	32	6		1	2
Spinal Muscular Atrophy	30	27	14		3	5,6,13
Myotonic dystrophy	29	26	5	2	1	3,11,1
Hurler syndrome	8	7	2	1		4
Spondyloepiphyseal dysplasia	7	6	1	1		12
Hemophagocytic lymphohistiocytosis	5	4	1	1		18
Monoamine oxidase A deficiency	5	4	2	1		10
X-linked MR	5	4		1		17
Paternity testing	3	2	2		1	19
Wilms tumors	3	2	1		1	15
Canavan disease	2	1	2		1	7
Nonsyndromic hearing loss	2	1		1		16
Nail-patella syndrome	1	0		1		8
Other diseases	271	271	56			

**Total**	**500**	**481**	**117**	**11**	**8**	

**Table 2:** List of nineteen abnormal karyotype results

Case no	Karyotype STC	Karyotype LTC	≥ 36 years	Karyotype AF	Follow-up testing	Type	Pregnancy follow-up and clinical outcome	DNA analysis or metabolic testing
**Aneuploidy in STC and LTC**								
1	47, XXY[3]	47, XXY[9]	no		-		TOP	not affected
2	FISH(13,18)×2, (X, Y)×1, (21)×3	47, XY, +21[8]	yes		-		TOP	not determined
3	47, XY, +21[2]	47, XY, +21[8]	no		-		TOP	not determined
4	47, XXY[4]	47, XXY[8]	no		-		TOP	not affected
5	47, XY, +18[3]	47, XY, +18[11]	yes		-		TOP	not determined
6	47, XY, +21[4]	47, XY, +21[8]	yes		-		TOP	not determined

**Aberration in STC, not in LTC**								
7	47, XX, +3[3]/46, XX[4]	46, XX[11]	yes		AF 46, XX	CPM I	continued	not affected
8	45, X[4]	46, XX[29]	no		AF 46, XX	CPM I	continued	not affected
9	47, XY, +15[2]/46, XY[2]	46, XY[29]	no		AF 46, XY, no UPD(15)	CPM I	continued	not affected
10	48, XY, +3, +21[5]/ 46, XY[6]	46, XY[16]	no		AF 46, XY	CPM I	continued	not affected

**Table 3:** List of nineteen abnormal karyotype results (continued)

Case no	Karyotype STC	Karyotype LTC	≥ 36 years	Karyotype AF	Follow-up testing	Type	Pregnancy follow-up and clinical outcome	DNA analysis or metabolic testing
**Aberration in LTC, not in STC**								
11	46, XX[4]	47, XX, +7[8]/46, XX[21]	no		-		TOP*	affected
12	46, XX[50]	92, XXXX[23]/46, XX[27]	no		-		partus 33 weeks, livebirth, no congenital anomalies	not affected
13	46, XY[4]	47, XY, +?18[2]/ 46, XY[37]	yes		-		unknown	affected
14	46, XX[8]	47, XX, +?20[2]/46, XX[7]	yes		-		TOP*	affected

**Aberration in STC and LTC**								
15	45, X[3]/46, XX[1]	45, X[1]/46, XX[8]	yes		skin biopsy post partum 46, XX	CPM III	TOP*	affected
16	45, X[1]/46, XY[2]	45, X[1]/46, XY[28]	no		-		continued, livebirth, no congenital anomalies	not affected
17	46, X, inv(Y)[5]	46, X, inv(Y)[9]	no		inv(Y)pat		TOP*	affected
18	47, XX, +mar[1]/ 46, XX[3]	47, XX, +mar[1]/ 46, XX[28]	no		parental karyotype normal		TOP	affected*

**Amniotic fluid sample**								
19	-	-	yes	45, X[10]/ 47, XXX[9]/ 46, XX[2]	karyotype blood post partum 45, X[9]/ 47, XXX[33]/46, XX[8], normal phenotype		continued, livebirth, no congenital anomalies	not affected

* TOP: termination of pregnancy based on affected pregnancy (positive DNA analysis or metabolic testing); CPM: confined placental mosaicism

In 4 of the aberrations present in STC but not in LTC, additional testing by amniocenteses showed a normal fetal karyotype and, therefore, the mosaicism in the CV samples appeared to be a confined placental mosaicism (CPM type I).

In 3 of the 4 aberrations present in LTC but not in STC, DNA mutation analyses were positive and the respective pregnancies were terminated. Therefore no cytogenetic follow-up testing was performed. In the 4^th ^case the percentage of tetraploid cells in the LTC was 46%. Although tetraploid cells are common in CV samples, particularly in LTC, this high percentage was a reason to report this finding and to continue pregnancy follow-up by ultrasound examination. The child was born pre-term at 33 weeks of pregnancy without congenital anomalies.

The abnormalities detected in both STC and LTC included two low-grade sex chromosome mosaicisms (cases 15 and 16). Case 15 appeared positive upon DNA mutation analysis. Therefore the pregnancy was terminated. In case 16 only 1 out of 4 metaphases in STC and 1 out of 28 metaphases in LTC showed a 45, X karyotype, all other metaphases were 46, XY. No follow-up amniocentesis was performed and the pregnancy was followed by ultrasound examination showing a normal male fetus. The 3^rd ^sample showed a paternally inherited 45, X, inv(Y) karyotype. The 4^th ^showed a marker chromosome in a single metaphase in both STC and LTC. The parental karyotypes were normal. The fetus appeared positive upon DNA mutation testing. The pregnancy was terminated.

Taken together, we conclude that karyotyping resulted in a diagnostic yield of 19 (3.8%) abnormal karyotypes, including 12 (2.4%) representing CV mosaicisms giving rise to uncertainty to prospective parents about the fetal prognoses.

## Discussion

In case an invasive prenatal test is performed for DNA mutation or metabolic testing, most prenatal centres offer traditional karyotyping (TK) as an additional test to exclude the presence of chromosome abnormalities. There is also no evidence from the literature that the prevalence of chromosomal abnormalities is higher in pregnancies with a referral for DNA mutation or metabolic analysis. The majority of the cases included in our current study represented CV samples (91.2%) because DNA isolation for mutation detection can be performed immediately after tissue sampling and a diagnostic result can be obtained in the first trimester. In contrast, AF cells have to be cultured first and, hence, a diagnostic result can only be obtained in the second trimester. In total, 19 (3.8%) abnormal karyotypes were denoted. When no cytogenetic testing was performed in the group of pregnant women < 36 years 11 (2.9%) cytogenetic aberrations would remained undetected. In 8 of these 11 it was uncertain whether the abnormal karyotype represented the true genetic constitution of the fetus due to mosaicisms. Mosaicisms are thought to be present in ~1% of CV samples, and have been confirmed in the fetus in 5-25% of these cases [18,19]. In our study population of 454 CV samples 12 (2.6%) showed a chromosome mosaicism with uncertain clinical impact. Uncertain diagnostic results are leading to parental anxiety and mostly require follow-up testing. For example in cases 9 and 11 (Tables 2 and 3), next to additional TK of amniotic fluid, additional DNA testing is required to exclude uniparental disomies (UPD) for chromosomes 7 and 15. Prenatal UPD(7) testing, however, is questionable considering the mild phenotype [20]. The risk of UPD(15) when trisomy 15 mosaicism has been detected, upon CV or AF analysis, has been estimated to range from 11% to 29% [21,22].

The overall percentage of mosaicism in our study (2.4%) score slightly higher as the 1-2% reported in the literature [18,19,23]. A possible explanation is the use of both the STC and LTC procedure. As individual laboratories use different protocols to examine CV samples (LTC only, STC combined with LTC or QF-PCR alone), the incidence of mosaicism is likely to vary to some degree between laboratories.

With the implementation of targeted molecular-cytogenetic tests such as MLPA and QF-PCR, the question arises whether TK should remain the gold standard or could be replaced by RAD for pregnancies at risk for common aneuploidies. RAD test results are unequivocal and available within 1-2 days, which considerably reduces parental anxiety [16]. In case of thalassaemia, for example, RAD has already shown to be the best approach for the detection of chromosomal abnormalities when prenatal invasive testing is performed [3]. Next to the possibility to recognise maternal cell contamination, RAD is also cost-effective particularly when performed large scale [24]. For pregnancies with an increased risk of Down syndrome, a change of policy from full karyotype analysis to rapid molecular aneuploidy testing would result in a failure to detect chromosome abnormalities that may have clinical consequences. This residual risk has been estimated to be 0.07% [25-27]. Next to the debate of targeted RAD replacing TK there is a discussion whether the scope of diagnostic testing should be broader than karyotyping. Although broadening the scope of testing benefits in terms of clinically relevant findings that would otherwise be missed, a serious challenge is that, with the present state of knowledge, results of such testing could be difficult to interpret [28], creating parental anxiety, uncertainty and unnecessary termination of pregnancy. Our overall tendency towards broadening the scope of prenatal testing is using genome-wide microarray analysis in high-risk pregnancies (e.g. with fetal ultrasound abnormalities) and narrowing (targeting) the scope in low-risk pregnancies.

In our prenatal diagnostic service, RAD (QF-PCR) is already implemented as replacement for the STC in all CV samples and offered as stand-alone test for pregnancies with an increased risk of Down syndrome, such as a positive screening test result or an advanced maternal age, as a test of choice. With the application of a cell dissociation protocol steps are taken to prevent fully discrepant results between cytotrophoblasts and mesenchymal core cells for the chromosomes 13, 18, 21, X and Y. Herewith, the cytotrophoblast cells (analyzed in STC) and mesenchymal core cells (analyzed in LTC) can be tested for RAD separately [29,30] and, thus, discrepancies as in cases 8 and 10 would have been detected if RAD were used. The application of RAD to replace karyotyping in both STC and LTC will prevent findings without clinical relevance and/or uncertain outcome.

Limitations of this retrospective study should be addressed. First, this study does not represent all pregnant women primary referred for molecular or metabolic testing. Some of the pregnancies were terminated because of a positive DNA mutation result and, therefore, the impact of the respective mosaicisms could not be evaluated. Secondly, although we already implemented QF-PCR as stand-alone test in our routine prenatal practice as a test of choice for pregnant women with an increased risk for Down syndrome, the QF-PCR test was not performed on the cases presented in this study.

Taken together, we conclude that there is additional diagnostic value of TK. However, there is also a need to limit uncertain test results as secondary findings. In this population with a low risk for a chromosomal aneuploidy, we recommend to implement QF-PCR as replacement of the STC and to give parents the autonomy to choose between karyotyping and QF-PCR.

The results of this study are in line with those reported by Tse et al [3], showing that RAD seems to be the best approach for the detection of chromosomal abnormalities when invasive prenatal testing is performed for the diagnosis of thalassaemia. The broader application of this study may create awareness and reconsideration of national standards for prenatal cytogenetic testing, a step towards international harmonized procedures and prenatal care.

## Competing interests

The authors declare that they have no competing interests.

## Authors' contributions

AK and AS drafted the manuscript. AS, BF and AK evaluated the cytogenetic results, LH the DNA diagnostic test results. AS, BF, LH, HZ and JP have critically reviewed and approved the manuscript. HZ clinically examined the pregnancy and performed the majority of the invasive procedures. IvdB referred and counselled most of the prospective parents and clinically examined the fetus. All authors read and approved the manuscript.

## List of abbreviations

AF: amniotic fluid; AMA: advanced maternal age; CPM: confined placental mosaicism; CV: chorionic villus; FISH: fluorescence in situ hybridization; LTC: long-term culture; MLPA: multiplex ligation-dependent probe amplification; NPDN: Network Prenatal Diagnostics Nijmegen; QF-PCR: quantitative fluorescent polymerase chain reaction; RAD: rapid aneuploidy detection; STC: short-term culture; TK: traditional karyotyping; UPD: uniparental disomy.
